# Porous Diaphragm Syndrome: A Literature Review

**DOI:** 10.7759/cureus.87138

**Published:** 2025-07-01

**Authors:** Mohsin Y Murshid, Anfal Nawawi, Hamdan S AlAmri, Abdulwudod M Hefdi, Abdulmalik M AlShamrani, Faisal AlNazawi

**Affiliations:** 1 General Surgery, Hera General Hospital, Makkah, SAU; 2 General Surgery, King Abdullah Medical Complex, Jeddah, SAU; 3 General Surgery, Al Hada Armed Forces Hospital, Taif, SAU; 4 General Surgery, Prince Mohammed Bin Nasser Hospital, Jizan, SAU; 5 General Surgery, International Medical Center, Jeddah, SAU

**Keywords:** diaphragmatic fenestration, hepatic hydrothorax, peritoneal dialysis, pleural effusion, porous diaphragm syndrome, vats

## Abstract

Porous diaphragm syndrome (PDS) is a rare and underrecognized condition characterized by the transdiaphragmatic migration of peritoneal contents into the pleural cavity through defects in the diaphragm, most often affecting the right hemidiaphragm. It is commonly associated with hepatic hydrothorax, peritoneal dialysis-related hydrothorax, and peritoneal carcinomatosis. Despite its anatomical simplicity and the availability of effective surgical treatment, PDS is frequently misdiagnosed, leading to repeated pleural drainage, inappropriate therapies, and treatment delays. This review synthesizes evidence from the past 25 years, drawing from PubMed, Scopus, and Web of Science, with a focus on clinical presentations, diagnostic methods, surgical management, and long-term outcomes. PDS most often presents as recurrent right-sided pleural effusion in cirrhotic or dialysis-dependent patients. Diagnostic approaches include pleural fluid analysis, CT peritoneography, peritoneal scintigraphy, and video-assisted thoracoscopic surgery (VATS), which also serves as the definitive therapeutic modality. VATS enables the direct visualization and repair of diaphragmatic defects with high success rates and low recurrence rates. Surgical correction often allows peritoneal dialysis to be resumed, while cirrhotic patients may benefit from transjugular intrahepatic portosystemic shunt (TIPS) or liver transplantation for long-term control. PDS should be considered in all cases of unexplained, recurrent pleural effusion in patients with underlying intra-abdominal pathology. Early recognition and multidisciplinary intervention, especially through thoracoscopic techniques, are key to improving outcomes and reducing recurrence.

## Introduction and background

Porous diaphragm syndrome (PDS) is a rare yet progressively acknowledged condition. The term porous diaphragm syndrome was introduced by Dr. Paul Kirschner, who eloquently described the anatomic defects that permit the passage of fluids, cells, tissue, and gases through diaphragmatic openings. As described in his landmark 1998 paper, he collectively referred to these clinical presentations as “porous diaphragm syndromes." It is defined by the translocation of fluid from the peritoneal cavity into the pleural space via diaphragmatic defects. These defects are often too small to be detected by conventional imaging techniques and are only identifiable during thoracoscopic inspection or intraoperative dye studies. The overall incidence of PDS remains unknown due to limited epidemiological data and the frequent underdiagnosis of this condition across its various clinical presentations.

The defects are typically situated in the central tendinous region of the diaphragm, facilitating unidirectional fluid movement due to pressure changes, resulting in clinically significant pleural effusions, predominantly on the right side [1]. The notion of diaphragmatic porosity was proposed in the 1960s but became more clinically significant with the identification of unexplained right-sided pleural effusions in cirrhotic patients and individuals undergoing peritoneal dialysis. In numerous patients, no apparent pulmonary or cardiac etiology was identified, and surgical exploration or advanced imaging frequently disclosed subtle diaphragmatic defects undetectable by standard imaging studies [2,3]. In reproductive-age females, thoracic endometriosis must be considered and excluded based on clinical history, hormonal correlation, or intraoperative findings.

PDS is primarily linked to hepatic hydrothorax and peritoneal dialysis-associated hydrothorax. Additional related conditions include chylothorax, urinothorax, and malignant effusions resulting from peritoneal carcinomatosis [4,5]. Early identification is essential to prevent recurrent thoracentesis, postponed definitive treatment, or erroneous management of presumed pulmonary conditions. The movement of fluid is facilitated by negative intrathoracic pressure, increased intra-abdominal pressure, and structural deficiencies in the diaphragm. These vulnerabilities may be congenital, acquired through chronic inflammation, or induced by pressure [6]. The increasing utilization of video-assisted thoracoscopic surgery (VATS) has enhanced the direct visualization of diaphragmatic fenestrations, which thereby confirmed the anatomical foundation of the syndrome and helped formulate treatment strategies [7].

This review endeavors to explore the pathophysiology, clinical manifestations, diagnostic evaluation, and management strategies of PDS, highlighting its significance in thoracic surgical practice. The review aims to cover the following key areas: the pathophysiologic mechanisms and anatomical substrates of PDS, strategies to improve diagnosis using current imaging and operative techniques, and the most effective and condition-specific treatment strategies, particularly from a thoracic surgical perspective.

## Review

Methods

This review was conducted to synthesize the current understanding of PDS across its various etiologies, diagnostic approaches, and management options. The literature search was performed using PubMed, Scopus, and Web of Science databases on April 3, 2025, by employing the following search terms: “porous diaphragm,” “pleuroperitoneal communication,” “hepatic hydrothorax,” “peritoneal dialysis hydrothorax,” “diaphragmatic fenestration,” and “catamenial pneumothorax.” Both MeSH terms and free-text searches were applied. Articles published between January 1990 and April 2025 were considered.

Inclusion criteria were English-language literature reviews, original research, case series, and relevant case reports involving human subjects. Studies not involving diaphragmatic communication or unrelated thoracic conditions were excluded. Two authors screened titles and abstracts independently, with full-text reviews performed for eligible papers. Discrepancies were resolved by discussion. Reference lists of key articles were also screened to identify additional sources. Due to the nature of the review, formal quality appraisal and quantitative meta-analysis were not conducted. Instead, included studies were grouped thematically based on their focus-such as pathophysiology, clinical manifestations, diagnostics, or management, and were analyzed narratively to identify recurring patterns, highlight condition-specific findings, and summarize current practices across different etiologies.

Pathophysiology

PDS is caused by congenital or acquired defects in the diaphragm, which predominantly affect the central tendinous region. This allows an abnormal connection between the peritoneal and pleural cavity, enabling the movement of peritoneal contents (such as ascitic fluid, dialysate, urine, chyle, or malignant cells) into the thoracic cavity, usually resulting in right-sided pleural effusions [8,9].

Diaphragmatic Structure and Vulnerability

The diaphragm comprises peripheral muscular areas and a central tendinous region, the latter being more prone to fenestration due to its relative thinness and poor blood supply [3]. The right hemidiaphragm is more frequently impacted in PDS, probably due to the lack of a protective organ such as the heart on the right side; direct hepatic pressure, which may worsen micro-tears or congenital apertures [3]; and embryonic fusion anomalies of the pleuroperitoneal membranes [10]. Congenital defects may signify enduring embryologic channels, whereas acquired pores are generally associated with chronic elevation of intra-abdominal pressure, inflammation, or trauma [11].

Pressure Gradient-Driven Migration

The negative intrathoracic pressure generated during inspiration contrasts sharply with positive intra-abdominal pressure, especially in conditions like ascites, peritoneal dialysis, or bowel obstruction. This pressure differential acts as a driving force that pulls fluid into the pleural space through diaphragmatic pores [3,11]. This explains why right-sided effusions are more common and why pleural fluid may rapidly reaccumulate following thoracentesis unless the peritoneal source is addressed [8].

Lymphatic Stomata and Fenestration Theory

The mesothelium of the diaphragm consists of lymphatic stomata, which are microscopic openings that facilitate the clearance of peritoneal fluid. These openings may become dilated or dysfunctional under pathological conditions, resulting in leakage of fluid or even cellular elements [12]. Experimental studies suggest that once these stomata or adjacent connective tissue fibers are disrupted, a one-way transdiaphragmatic flow may occur, which allows peritoneal contents to accumulate in the pleural space [10].

Histologic and Surgical Correlates

Histologically, diaphragmatic fenestrations appear as pinpoint gaps lined with attenuated mesothelium or granulation tissue [3]. These defects are too small to be visualized by standard imaging but are often identified intraoperatively during VATS, especially when methylene blue or indocyanine green (ICG) is instilled into the peritoneal cavity to highlight the leak [12].

Contributing Pathologies

PDS often occurs in the settings of conditions that result in chronic elevation of intra-abdominal pressure, including cirrhosis with ascites, peritoneal dialysis, malignancy with peritoneal carcinomatosis, and retroperitoneal urinoma or lymphatic leakage [3,8,11]. Repeated pressure on the diaphragm in these settings likely exacerbates existing weaknesses or creates new fenestrations over time.

Clinical manifestations

PDS encompasses a spectrum of clinical scenarios most often resulting in recurrent or persistent right-sided pleural effusions. The manifestations depend on the underlying pathology, the volume and nature of peritoneal fluid, and whether the communication is transient or persistent [1,8].

Hepatic Hydrothorax

The most common and well-studied cause of PDS is hepatic hydrothorax, seen in approximately 5-10% of patients with advanced cirrhosis and portal hypertension. It often presents in the absence of clinically significant ascites [13]. These patients develop recurrent transudative pleural effusions, commonly on the right side as a result of direct communication between the peritoneal cavity and pleural space through diaphragmatic fenestrations [14]. Clinical presentations include Progressive dyspnea, Orthopnea, Non-productive cough, and decreased exercise tolerance. While plain chest radiographs may reveal a large pleural effusion, definitive diagnosis often requires advanced imaging and sometimes direct visualization during thoracoscopy [14].

Hydrothorax Associated With Peritoneal Dialysis

In patients receiving continuous ambulatory peritoneal dialysis (CAPD), PDS may lead to a rapid onset of unilateral pleural effusion, usually appearing within the initial six months of dialysis initiation. Incidence rates vary from 1.6% to 10%, and unrecognized effusion frequently results in respiratory distress and the discontinuation of dialysis [11,15]. Noteworthy features of hydrothorax include right-sided pleural effusion and elevated glucose levels in pleural fluid similar to dialysate. Rapid reaccumulation following thoracentesis and resolution upon discontinuation of peritoneal dialysis. Early diagnosis is crucial, as continuing dialysis amid diaphragmatic leakage can worsen the effusion or lead to empyema associated with hydrothorax [11].

Chylothorax, Urinothorax, and Malignant Pleural Effusions

Less common but important manifestations of PDS include: (i) Chylothorax, in which chyle translocates through diaphragmatic defects from the peritoneum to the pleural cavity. This can occur in lymphatic obstruction or peritoneal carcinomatosis [16]. (ii) Urinothorax, where urine leaks into the pleural space from retroperitoneal rupture or obstructive uropathy [17]. (iii) Malignant pleural effusions secondary to peritoneal carcinomatosis-notably in ovarian, gastric, or peritoneal mesothelioma-where tumor cells gain access to the thoracic cavity through fenestrations [18]. (iv) In these cases, effusions may be exudative, and cytology can help in establishing the underlying etiology. Diagnosis requires a combination of imaging, pleural fluid analysis, and often diagnostic thoracoscopy.

Catamenial Pneumothorax

Catamenial pneumothorax is a distinct clinical condition that is similar to PDS, notably with its pathophysiological process related to diaphragmatic fenestrations. It usually manifests in premenopausal women within 72 hours following menstruation. It is regarded as the most prevalent sign of thoracic endometriosis syndrome (TES). [19] The disease predominantly impacts the right hemidiaphragm. Numerous little fenestrations or defects are typically detected intraoperatively, usually during VATS. These abnormalities facilitate the transdiaphragmatic movement of air, endometrial tissue, or blood. The outcome is recurrent right-sided pneumothorax [20].

Histopathology shows glandular or stromal endometrial cells within the pleural or diaphragmatic tissue. Diagnosis is mainly clinical, based on the timing of menstruation, recurrence, and intraoperative observations [21]. Management includes thoracoscopic excision or ablation of discernible endometrial implants. Diaphragmatic deficiencies can also be surgically corrected if indicated, frequently utilizing mesh reinforcement. Chemical pleurodesis is frequently conducted to diminish recurrence. Postoperative hormonal suppression is generally advised, utilizing medications such as gonadotropin-releasing hormone (GnRH) analogs or oral contraceptives [22,23]. Catamenial pneumothorax is a gynecological variation of primary spontaneous pneumothorax. The acknowledgment of this condition is crucial for facilitating multidisciplinary therapy and avoiding misdiagnosis in women experiencing recurrent pneumothorax.

Other Rare Presentations

In rare cases, PDS can present with: (i) bilateral effusions (in advanced cirrhosis or peritoneal malignancy); (ii) pneumothorax or hydro-pneumothorax if air enters the pleural space from bowel perforation; (iii) herniation of bowel or omentum through large defects, mimicking diaphragmatic hernia [24]; (iv) these atypical presentations can be misleading and are often misdiagnosed as primary pulmonary conditions, delaying appropriate intervention. The spectrum of clinical presentations and associated mechanisms across various etiologies is summarized in Table 1.

**Table 1:** Clinical presentation of porous diaphragm syndrome CAPD: continuous ambulatory peritoneal dialysis; PD: peritoneal dialysis

Underlying condition	Mechanism of pleural effusion	Laterality	Characteristics
Cirrhosis (hepatic hydrothorax)	Ascitic fluid migration via diaphragmatic fenestrations	Right > left	No infection; transudate
Peritoneal dialysis	Dialysate migration through microdefects	Right > left	High pleural glucose; often early in PD
Peritoneal carcinomatosis	Tumor cell migration via pores	Bilateral/right	Often exudative; cytology may be positive
Chylous ascites	Chyle migration under high pressure	Right or bilateral	Milky pleural fluid; ↑ triglycerides
Urinothorax	Retroperitoneal urine leakage through pores	Right	Pleural fluid: low pH, ↑ creatinine
CAPD-related hydrothorax	Repeated dialysate leakage into the pleural space	Right	Often resolves after peritoneal rest

Diagnostic evaluation

**Figure 1:**
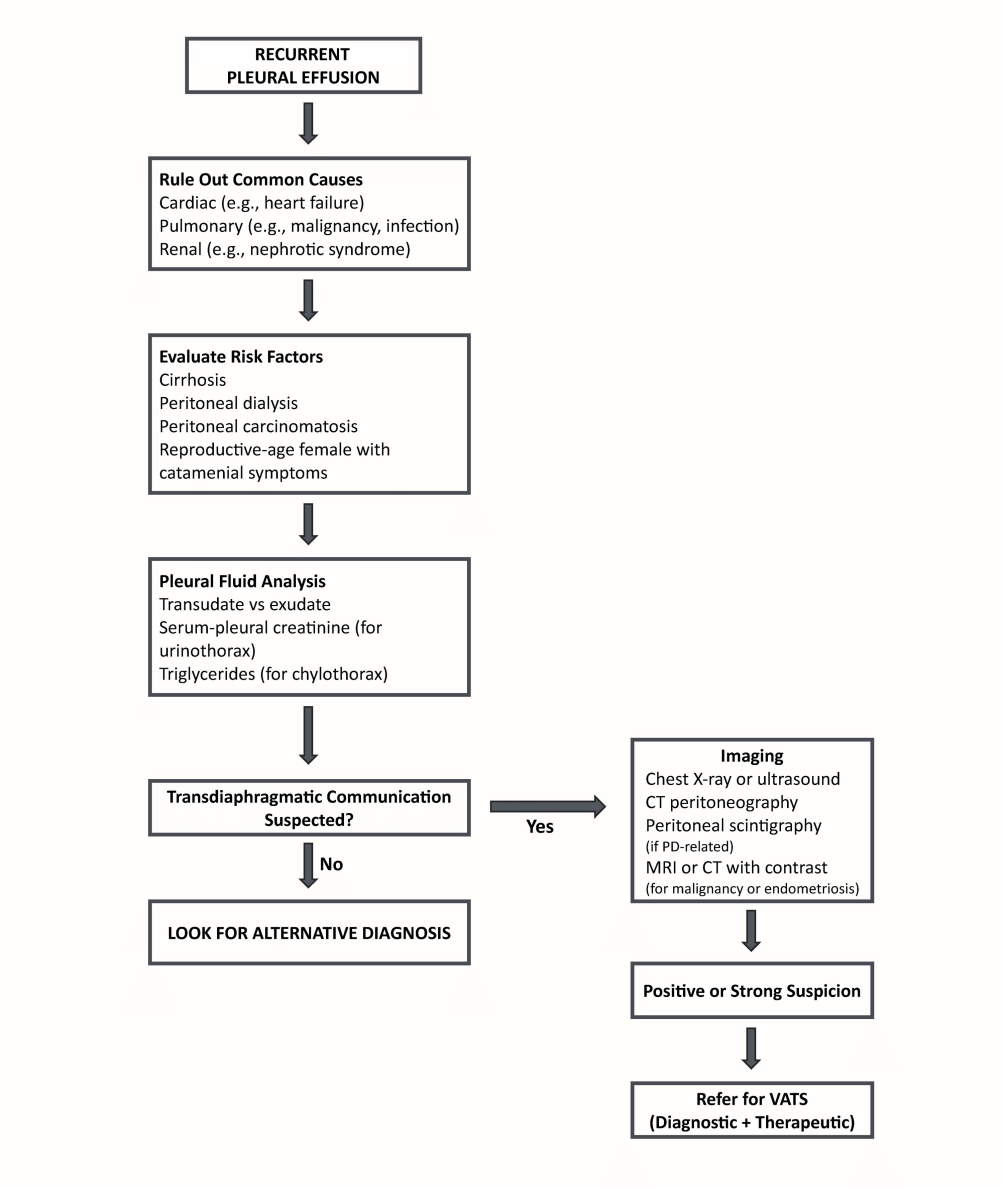
Diagnostic approach to porous diaphragm syndrome CT: computed tomography; MRI: magnetic resonance imaging; PD: peritoneal dialysis; VATS: video-assisted thoracoscopic surgery This is an original figure created by the authors

Diagnosing PDS requires a high level of suspicion, particularly in patients presenting with unexplained right-sided pleural effusions associated with recognized intra-abdominal conditions such as cirrhosis or peritoneal dialysis. Diagnostic difficulty emerges from the microscopic nature of diaphragmatic defects, which usually evade detection through standard imaging techniques. A multimodal approach integrating clinical context, biochemical analysis, imaging, and, on occasion, surgical exploration is crucial. The suggested diagnostic pathway is outlined in Figure 1.

Clinical Indicators and Patient Selection

Notable clinical features that suggest PDS include recurrent right-sided pleural effusion in the absence of identifiable cardiac or pulmonary pathology, minimal or absent ascites despite the presence of a significant pleural effusion, and the development of a new pleural effusion following the initiation of peritoneal dialysis. Additional indicators include resolution of ascites after thoracentesis and rapid reaccumulation of pleural fluid shortly after drainage [14,25]. When these findings are observed, further targeted diagnostic investigations should be initiated to confirm the presence of pleuroperitoneal communication.

Pleural Fluid Analysis

Pleural fluid analysis provides valuable clues in the diagnosis of PDS. Although not diagnostic alone, pleural fluid characteristics guide further investigation. Biochemical analysis, though non-specific, often strongly suggests communication with the peritoneal cavity. Table 2 shows characteristics of pleural fluid in various PDS-related conditions.

**Table 2:** Pleural fluid characteristics in porous diaphragm syndrome-related effusions LDH: lactate dehydrogenase

Etiology	Appearance	Transudate/exudate	Key biochemical findings
Hepatic hydrothorax	Clear	Transudate	Low protein, low LDH
Peritoneal dialysis leak	Clear	Transudate	High glucose; resembles dialysate
Chylothorax	Milky	Exudate	Triglycerides >110 mg/dL
Urinothorax	Straw/pale	Transudate	pH <7.4, pleural creatinine > serum creatinine
Malignant effusion	Bloody	Exudate	Positive cytology, high LDH

Imaging Modalities

Chest X-ray and ultrasound: Chest radiographs generally show right-sided pleural effusions but are unable to identify the etiology [1]. Ultrasound aids in the identification of free fluid, assists in guiding thoracentesis, and evaluates diaphragmatic motion; however, it is incapable of detecting microdefects.

CT-scan: Contrast-enhanced CT of the thorax and abdomen may reveal peritoneal fluid collections, diaphragmatic hernias or large defects, and associated hepatic, renal, or neoplastic pathology. CT peritoneography, which involves the intraperitoneal administration of contrast, offers greater sensitivity. It can demonstrate the presence of contrast material within the pleural space, thereby confirming a transdiaphragmatic communication [26].

Peritoneal scintigraphy: This is the most specific noninvasive test for establishing pleuroperitoneal communication. It employs technetium-99m sulfur colloid administered into the peritoneal cavity. A positive result is confirmed by the presence of the radioisotope in the pleural space, generally within two to six hours. [27]

MRI: MRI provides exceptional soft tissue resolution; however, its utility in detecting fenestrations is limited, unless diaphragmatic tumors are suspected [4].

Thoracoscopic confirmation: VATS is the gold standard for the diagnosis of PDS when noninvasive tests are inconclusive. It allows direct visualization of fenestrations, which are often seen as tiny pores in the central tendon. Dye testing using intraperitoneal methylene blue or ICG, which helps localize the leak during surgery [12] and simultaneous surgical repair and pleurodesis, if needed. In some centers, intraoperative CO₂ insufflation of the abdomen can demonstrate bubble passage into the pleural space.

Laparoscopy: Laparoscopy has limited diagnostic application but may be useful when abdominal exploration is indicated (e.g., for tumor staging or hernia repair). Its ability to detect superior diaphragmatic defects is inferior to thoracoscopy, as it visualizes the peritoneal surface and not the pleural surface [28].

Emerging modalities: Fluorescence thoracoscopy using ICG injected into the peritoneal cavity shows promising results. ICG binds to plasma proteins and leaks through these defects in the diaphragm, appearing as fluorescent streams under near-infrared imaging [12]. It enhances the detection of subtle or occult fenestrations, especially useful in minimally invasive surgery. Table 3 shows a summary of various modalities that aid in the diagnosis of PDS.

**Table 3:** Diagnostic modalities for porous diaphragm syndrome CT: computed tomography; VATS: video-assisted thoracoscopic surgery

Modality	Utility	Sensitivity/specificity	Notes
Chest X-ray	Detects effusion	Low/low	Cannot identify diaphragmatic defects
Ultrasound	Assesses pleural fluid; guides thoracentesis	Moderate/low	May show diaphragmatic motion
CT scan	Detects hernia, ascites, and gross defects	Moderate/low	Standard CT often misses microdefects
CT peritoneography	Shows contrast migration	Moderate/high	Requires intraperitoneal contrast
Peritoneal scintigraphy	Demonstrates pleuroperitoneal communication	Moderate–high/very high	Gold standard noninvasive test
VATS	Direct visualization of defects	Very high/very high	Diagnostic and therapeutic
Fluorescence-guided thoracoscopy	Enhances visualization intraoperatively	High/high (experimental)	Useful in subtle/occult defects

Management

Treatment must be personalized depending on the underlying cause, severity of symptoms, recurrence pattern, and patient's functional ability. It involves various non-surgical (conservative) and surgical interventions, generally progressing from medical therapy to definitive surgical repair.

Conservative Treatment

Hepatic hydrothorax: In patients with cirrhosis, the primary treatment modalities consist of sodium limitation, diuretics (commonly spironolactone and furosemide), and repeated therapeutic thoracentesis, if warranted [13]. Although thoracentesis can provide symptomatic relief, it fails to rectify the underlying defect and may elevate the risk of infection or pneumothorax. The transjugular intrahepatic portosystemic shunt (TIPS) procedure can improve portal hypertension and ascites, thereby decreasing hydrothorax. 50-70% of patients demonstrated clinical improvement following the procedure [29]. In refractory cases, liver transplantation serves as the definitive treatment, resulting in the alleviation of portal hypertension and the subsequent spontaneous closure of diaphragmatic fenestrations [14].

Hydrothorax associated with peritoneal dialysis: In patients undergoing peritoneal dialysis (PD), conservative measures include temporary cessation of PD with a transition to hemodialysis, decreasing dialysate volume and employing supine exchanges to mitigate intra-abdominal pressure, and implementation of peritoneal rest for a duration of two to six weeks to facilitate spontaneous closure. [15] Nevertheless, chronic hydrothorax frequently necessitates surgical intervention if CAPD is to be maintained. [30]

Pleurodesis: Chemical pleurodesis utilizing agents like talc, tetracycline, or bleomycin seeks to eradicate the pleural space and inhibit fluid accumulation. Success rates are elevated in PD-related hydrothorax up to 70%, whereas they are reduced and frequently transient in hepatic hydrothorax as a result of sustained elevated intra-abdominal pressures [31]. Furthermore, pleurodesis is relatively contraindicated for transplant candidates, as it may complicate subsequent surgical procedures.

Surgical Management

Surgical repair of diaphragmatic defects is the definitive intervention for cases that are recurrent, refractory, or characterized by high output.

VATS: VATS is the preferred approach due to its minimally invasive nature and high success rates. This procedure enables direct visualization of diaphragmatic fenestrations, which can be closed using nonabsorbable sutures. In cases involving multiple or large defects, reinforcement with biological mesh or prosthetic patches is often employed. Additionally, mechanical or chemical pleurodesis may be performed during the same procedure to reduce the risk of recurrence [7]. VATS repair has demonstrated success rates exceeding 80% in most studies, with many patients able to resume peritoneal dialysis within four to six weeks following surgery [32].

Laparoscopic and combined approaches: Laparoscopy may be beneficial when intra-abdominal access is required, particularly in cases involving concomitant hernia repair, oncologic exploration, or when thoracic surgery is contraindicated [28]. However, its utility in visualizing superior diaphragmatic defects is limited. Furthermore, laparoscopic-only repair has been associated with a higher recurrence rate compared to thoracoscopic or combined approaches.

Open thoracotomy: This method is reserved for complex or recurrent cases where VATS has failed or is not feasible due to adhesions or extensive disease. Though effective, open repair carries greater morbidity and longer recovery time [33].

Timing and Postoperative Considerations

**Figure 2:**
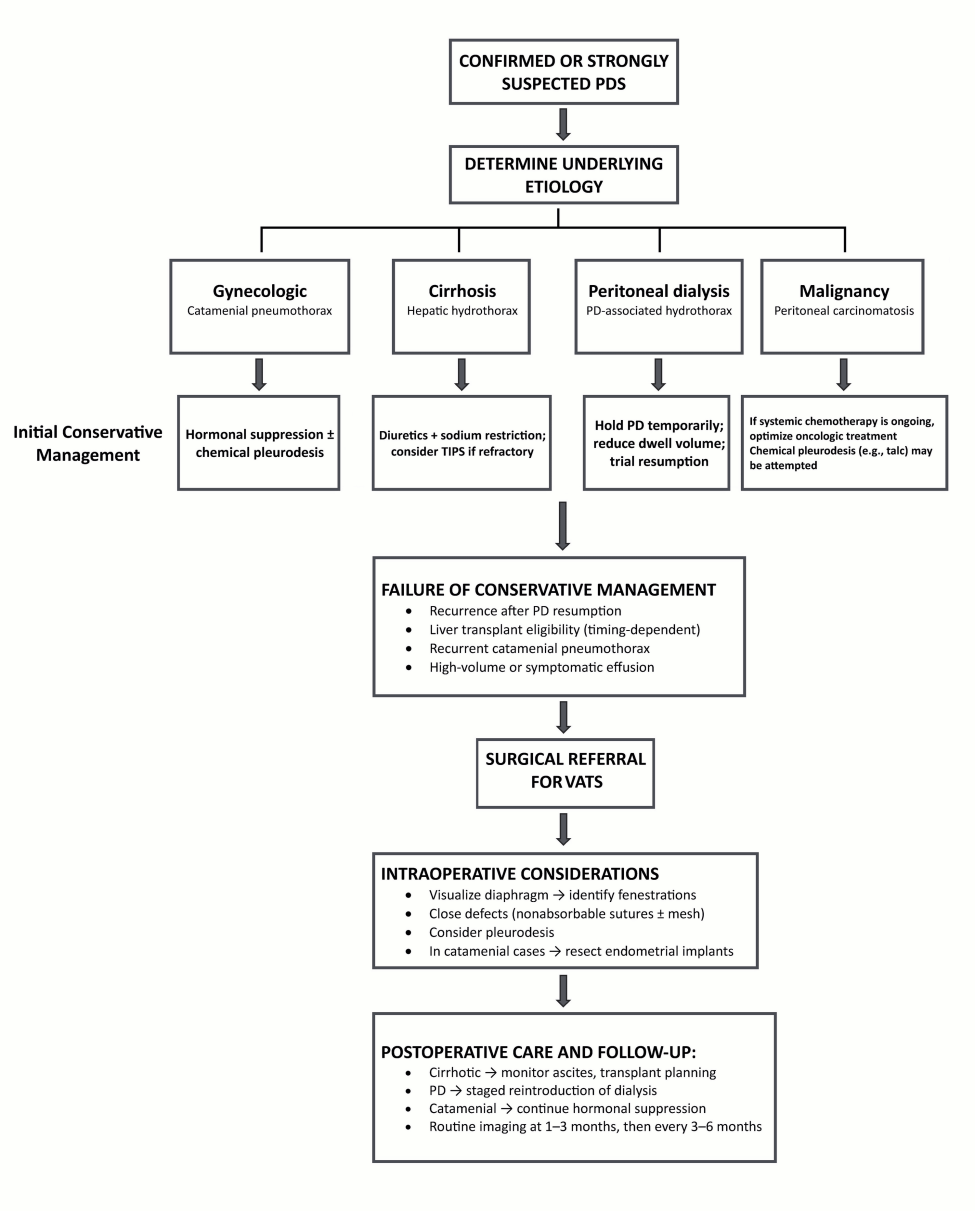
Management algorithm for confirmed or strongly suspected porous diaphragm syndrome PDS: porous diaphragm syndrome This is an original figure created by the authors

In PD patients, early surgical intervention may allow safe resumption of dialysis. In cirrhotic patients, surgical options should be weighed against transplant eligibility. Postoperative imaging or pleural drainage may be necessary to monitor recurrence. In some cases, a combined VATS + TIPS approach has been proposed for complex hepatic hydrothorax [34]. Figure 2 shows a management algorithm for confirmed or suspected cases of PDS. Table 4 summarizes various treatment modalities based on the etiology of PDS.

**Table 4:** Management strategies based on etiology CAPD: continuous ambulatory peritoneal dialysis; PDS: porous diaphragm syndrome; TIPS: transjugular intrahepatic portosystemic shunt; VATS: video-assisted thoracoscopic surgery

Etiology	First-line treatment	Definitive management	Notes
Hepatic hydrothorax	Diuretics, salt restriction	TIPS or liver transplantation	Pleurodesis is not ideal if transplant candidate
Peritoneal dialysis leak	Peritoneal rest, ↓ fill volume	VATS repair	CAPD may resume post-repair
Malignancy-related PDS	Treat the underlying cancer	VATS + pleurodesis ± mesh	Often palliative
Urinothorax	Relieve obstruction	Usually resolves with urologic intervention	Rarely requires surgical repair
Recurrent symptomatic cases	Multiple thoracenteses	VATS closure ± pleurodesis/mesh	Mesh use reduces recurrence

Prognosis and follow-up

The prognosis primarily depends on the underlying etiology and the success of definitive treatment. The presence of comorbidities is also a contributing factor. The condition itself is benign and treatable. A delay in diagnosis or failing to consider the underlying cause may lead to considerable morbidity, which includes recurrent hospitalizations, respiratory dysfunction, and loss of peritoneal dialysis access.

Natural History and Risk of Recurrence

If left untreated, PDS may lead to persistent pleural effusions, progressive restrictive respiratory symptoms, and an increased risk of empyema due to repeated thoracentesis and chronic pleural inflammation. Long-standing fluid accumulation may also contribute to pleural fibrosis. Hepatic hydrothorax tends to recur frequently due to persistent ascites and portal hypertension. One study noted that over 70% of cirrhotic patients with hepatic hydrothorax experienced at least one recurrence within six months of diagnosis when managed conservatively [31]. Spontaneous resolution of PDS in patients undergoing peritoneal dialysis is rare, particularly if CAPD continues. In such cases, recurrence rates may exceed 80% in the absence of surgical correction [15].

Outcomes After Surgical Repair

Surgical intervention yields excellent long-term outcomes. VATS closure of diaphragmatic defects has shown success rates exceeding 80%, with over 70-90% of patients able to resume CAPD following repair. Postoperative complications are infrequent, especially in elective cases [7,32,35]. Recurrence after surgery is typically linked to incomplete closure of diaphragmatic defects, the use of absorbable materials or sutures, or persistently elevated intra-abdominal pressure resulting from uncontrolled ascites or inappropriate dialysis parameters. The use of mesh reinforcement and adjunctive pleurodesis may help reduce the likelihood of recurrence.

Outcomes of Liver Transplantation

Liver transplantation provides an ideal long-term outcome for cirrhotic patients with hepatic hydrothorax. Generally, diaphragmatic fenestrations spontaneously close after transplantation as ascites resolves, and survival rates are comparable to those of other transplant indications [35]. Surgical pleurodesis is contraindicated in transplant candidates to avert future adhesions [14,29].

Post-treatment Monitoring

Patients undergoing treatment for PDS require individualized follow-up strategies tailored to the underlying etiology and treatment modality. Clinical surveillance should focus on symptoms suggestive of recurrence, such as dyspnea, cough, or reduced peritoneal dialysis efficacy. Regular chest imaging is advised to detect early reaccumulation of pleural fluid. In patients on peritoneal dialysis, reassessment of dialysate fill volumes, dwell times, and abdominal pressure tolerance is essential to minimize recurrence risk. For patients with hepatic hydrothorax, continued management of ascites and sodium intake, along with timely evaluation for liver transplantation, remains critical for long-term control.

Quality of Life and Functional Rehabilitation

The effective management of PDS can restore respiratory function, dialysis viability, and patient independence, and decrease hospitalizations. In contrast, undiagnosed or inaccurately diagnosed PDS substantially contributes to functional deterioration, especially in elderly or frail individuals [36]. Table 5 summarizes the outcomes and recurrence rate of PDS based on its various etiologies.

**Table 5:** Outcomes and recurrence rates of PDS by etiology PD: peritoneal dialysis; PDS: porous diaphragm syndrome; VATS: video-assisted thoracoscopic surgery

Etiology	Typical recurrence without surgery	Success rate after VATS repair	Recommended follow-up interval
Hepatic hydrothorax (cirrhosis)	>70% within 6 months	70–85%	Every 3–6 months or transplant clinic
Peritoneal dialysis–related hydrothorax	>80% upon resumption of PD	85–90% (with return to PD in most cases)	Weekly PD reassessment for 4 weeks, then monthly
Malignancy (peritoneal carcinomatosis)	High recurrence unless malignancy is controlled	~60–70% if the underlying disease is addressed	Imaging every 3 months; monitor pleural symptoms
Chylothorax (lymphatic leak)	Variable; depends on the underlying lymphatic repair	~70% with thoracic duct ligation or repair	Every 3–6 months post-intervention
Catamenial pneumothorax	Up to 50–60% if untreated	>85% when combined with hormonal suppression	3-monthly gynecology and thoracic review

## Conclusions

PDS is a clinically significant but underrecognized cause of recurrent pleural effusions, particularly in patients with cirrhosis, PD, or peritoneal malignancy. Early recognition requires a high index of suspicion, especially in cases of right-sided effusion with minimal ascites. While imaging can support the diagnosis, VATS remains the gold standard for both identification and repair of diaphragmatic defects. Timely surgical intervention can restore dialysis viability and prevent repeated hospitalizations. Ongoing research should aim to standardize diagnostic pathways, refine surgical techniques, and promote multidisciplinary coordination for optimal patient outcomes.
